# The Association of Polymorphisms in Base Excision Repair Genes with Ovarian Cancer Susceptibility in Chinese Women: A Two-Center Case-Control Study

**DOI:** 10.7150/jca.49925

**Published:** 2021-01-01

**Authors:** Mingyao Zhang, Zhiguang Zhao, Sailing Chen, Zongwen Liang, Jiawei Zhu, Manman Zhao, Chaoyi Xu, Jing He, Ping Duan, Anqi Zhang

**Affiliations:** 1 Department of Obstetrics and Gynecology, The Second Affiliated Hospital and Yuying Children's Hospital of Wenzhou Medical University, Wenzhou 325027, Zhejiang, China.; 2 Department of Pathology, The Second Affiliated Hospital and Yuying Children's Hospital of Wenzhou Medical University, Wenzhou 325027, Zhejiang, China.; 3 Department of Pediatric Surgery, Guangzhou Institute of Pediatrics, Guangzhou Women and Children's Medical Center, Guangzhou Medical University, Guangzhou 510623, Guangdong, China.

**Keywords:** BER, DNA repair, ovarian cancer, susceptibility, polymorphism

## Abstract

Base excision repair (BER) acts upon the most important mechanism of the DNA repair system, protecting DNA stability and integrity from the mutagenic and cytotoxic effects. Multiple researches have indicated that single-nucleotide polymorphisms (SNPs) in the BER-related gene may be associated with the susceptibility of ovarian cancer. However, the results are controversial. In this two-center case-control study, 19 potentially functional SNPs in six BER-related genes (*hOGG1*, *APE1*, *PARP1*, *FEN1*, *LIG3* and *XRCC1*) was genotyped in 196 ovarian cancer cases and 272 cancer-free controls. And, their associations with ovarian cancer risk were assessed by unconditional logistic regression analyses. We found that *PARP1* rs8679 and *hOGG1* rs293795 polymorphisms were associated with a decreased risk of ovarian cancer under dominant model (adjusted OR=0.39, 95% CI=0.17-0.90, *P*=0.026; and adjusted OR=0.36, 95% CI=0.13-0.99, *P*=0.049, respectively). Stratification analysis demonstrated that this association was more pronounced in the subgroups of lower BMI and patients with early menarche and serous carcinoma. Moreover, *LIG3* rs4796030 AA/AC variant genotypes performed an increased risk of ovarian cancer under recessive model (adjusted OR=1.54, 95% CI=1.01-2.35, *P*=0.046), especially in the subgroups of higher BMI, early clinic stage and the carcinoma at the left. These results suggested that *PARP1*, *hOGG1* and* LIG3* polymorphisms might impact on the risk of ovarian cancer. However, more researches with larger and different ethnic populations are warranted to support our findings.

## Introduction

Ovarian cancer is the main cause of death in gynecological malignancy according to global cancer statistics in 2018, with an estimated 184,799 deaths worldwide 1. The common risk factors of that result in extremely poor 5-year survival, such as asymptomatic characteristics and the lack of early detection strategies, have become an immediate problem to be solved for ovarian cancer 2, 3. Thus, ovarian cancer remains a great burden and challenge for the affected family and public health.

Numerous studies have indicated that the risk of ovarian cancer was related to *BRCA1* and *BRCA*2 genes. Marc D. *et al.* 4 found that *BRCA1*/*2* mutations may ultimately impact ovarian cancer mortality. Adrianna *et al.* 5 demonstrated significantly increased *APC* rs11954856 and rs351771 frequencies in Polish women with ovarian cancer. However, several genetic studies published recently had suggested that the risk of ovarian cancer was related to the mutations and polymorphic variants in DNA repair genes 6, 7. DNA repair mechanism mainly include direct repair, base excision repair (BER), nucleotide excision repair (NER) and mismatch repair (MMR) 8. The primary DNA damage repair pathway, BER, is responsible for the modification of the most common forms of DNA damage, such as spontaneous hydrolytic, oxidative and alkylative lesions and single-strand breaks 9. Although considerable genetic studies demonstrated that the deficiency of BER related genes would influence on ovarian cancer risk, the real relationship between both of them remained controversial 10.

BER processes can be divided into the following three basic steps: damage recognition/strand scission, gap tailoring and gap filling/nick ligation 11. Based on different types of base lesions, either of two subways (short patch or long patch BER) will be initiated by corresponding lesion-specific DNA glycosylase 12. 8-oxoguanine glycosylase (hOGG1) acts as one of the most important bifunctional glycosylases, which cannot only excise the substrate base but also incises DNA 3' to the damage site via an intrinsic AP lyase activity 13. Meanwhile, apurinic endonuclease 1 (APE1) removes the resulting 3'-obstructive termini and prepares for next polymerization and/or nick ligation 14. Differing from the classical initial step, however, the single-strand break repair (SSBR) depends on poly (ADP-ribose) polymerase 1 (PARP1) to recognize the damage and recruit other protein to the damage site 15. In addition, flap endonuclease (FEN1) was also included in our study for its gap-tailoring properties in the DNA ends 16. DNA ligase 1 (LIG1) and the complex of DNA ligase 3 (LIG3)/X-ray repair cross-complementing 1 (XRCC1) exhibit similar performance of nick ligation, but we only selected LIG3/XRCC1 as the investigated biomarker for its unique character of collecting proteins at the damaged site 17, 18. In the current study, we analyze the associations between 19 SNPs of six BER-related genes (*hOGG1*, *APE1*, *PARP1*, *FEN1*, *LIG3* and *XRCC1*) and ovarian cancer risk by genotyping method in 196 patients and 272 controls.

## Materials and Methods

### Patients and controls

In this hospital-based case-control study, 196 ovarian cancer patients were newly diagnosed and histopathologically confirmed in two different institutions of the Second Affiliated Hospital and Yuying Children's Hospital and the First Affiliated Hospital of Wenzhou Medical University (WMU) from February 2007 to March 2018. Additionally, we selected 272 cancer-free women who participated in the routine physical examination of the Second Affiliated Hospital and Yuying Children's Hospital of WMU. All of them were frequency-matched to cases on age (± 2 years) and race/ethnicity, and reconfirmed without any personal tumor history or family tumor history. All people included in the present research had signed a written informed consent. The demographic data and environmental exposure factors, such as age, BMI, menarche, menopause, number of pregnancies, were collected from each participant. Clinical and pathological information were further extracted from the patients' electronical database, including pathologic classification (WHO 2014), histology grade, FIGO stage (International Federation of Gynecology and Obstetrics), tumor size (the largest tumor diameter of the primary tumor) and anatomic neoplasm subdivision. However, the patients who were diagnosed without histology grade would be categorized as a single group of “undermined”. And, some patients with some rare pathological classification were included in the “others” pathological group. The research was approved by the Second Affiliated Hospital and Yuying Children's Hospital of WMU. Our study was conducted following the Declaration of Helsinki, and participants or guardians were required to sign informed consent forms. Blood samples were obtained from cases before receiving radiotherapy or chemotherapy.

### SNP selection and genotyping

The SNPs were selected from the NCBI dbSNP database (http://www.ncbi.nlm.nih.gov/projects/SNP) and the International HapMap Project database (http://hapmap.ncbi.nlm.nih.gov/) based on the following criteria: (1) located at the coding sequence, the 5' near gene, 5'-untranslated region (UTR), 3' UTR and 3' near gene, (2) minor allele frequency (MAF) of at least 5 % in Chinese populations reported in HapMap, (3) low linkage disequilibrium (LD) between SNPs with an r^2^ threshold of < 0.8, and (4) predicted SNPs potential function using the SNP function prediction (FuncPred) software (https://snpinfo.niehs.nih.gov/snpinfo/snpfunc.html). As a result, a full list containing 19 selected SNPs was presented with potential function in **Supplemental Table S1** 19.

For all cases, the DNA genomic was extracted from paraffin-embedded tissue using TIANquick FFPE DNA Kit (Qiagen Inc., Valencia, CA), while the DNA genomic of all controls were extracted from the peripheral blood specimens using the TIANamp Blood DNA Kit (TianGen Biotech Co. Ltd.). DNA purity and concentration were measured by a UV spectrophotometer (Nano Drop Technologies, Inc., Wilmington, DE).

According to standard protocols, the genotyping of 19 selected SNPs was performed with a TaqMan real-time PCR assay in ABI Prism 7900HT genetic detection system (Applied Biosystems Inc., USA) 20-22. To confirm the accuracy of the genotyping, 5% samples were randomly selected as positive controls (repeat samples) and negative controls (without DNA template). As a result, the genotyping success rate reached 96.8%, and the results of duplicated samples were 100% concordant.

### Statistical analysis

All statistical tests were performed by SAS software (Version 9.4; SAS Institute, Cary, NC, USA). Differences in demographic (e.g., age and BMI) and other covariates (e.g., menarche, menopause and number of pregnancies status) between patients and controls were evaluated by Pearson's χ^2^ test. To assess the associations of these 19 SNPs with ovarian cancer risk, the adjusted odds ratios (ORs) and 95% confidence intervals (CIs) were calculated for dominant (AB+BB vs. AA) and recessive models (BB vs. AB+AA) by unconditional logistic regression analyses. A and B respectively represent different alleles (wild and mutant) at this location. Additionally, stratification analysis was performed to further evaluate the significant associations existed in which demographic or clinic covariates groups. Deviation from Hardy-Weinberg Equilibrium (HWE) in the controls was assessed by a goodness-of-fit χ^2^ test. All *P* value was two-sided with a significant level < 0.05.

## Results

### Population characteristics

In the current study, we enrolled 196 ovarian cancer patients with an average age of 50.01 ± 12.51 years and 272 cancer-free controls with an average age of 50.48 ± 11.98 years. The demographic and clinical characteristics of all participants were shown in **Supplemental Table S2**. There were no significant differences between cases and controls in age (*P*=0.570), BMI (*P*=0.947), menarche (*P*=0.072), menopause (*P*=0.421) and number of pregnancies (*P*=0.448). To discuss the pathological classification, 132 (67.4%), 27 (13.8%) and 15 (7.7%) cases were subdivided into serous, mucinous and clear cell carcinoma, respectively, while 22 (11.2%) cases were included into “others” because of the limited availability of tumor samples. Regarding anatomic neoplasm subdivision, 27 (13.8%), 74 (37.8%) and 95 (48.5%) ovarian cancers occurred in the left, right and both sides, respectively. In term of tumor size, 27 (13.8%) patients reported with a tumor of < 5cm, 74 (37.8%) with a tumor of 5-10 cm and 95 (48.5%) with a tumor of >10cm. According to the FIGO stage, 96 (49.0%) and 100 (51.0%) cases distributed into the stage of I+II and III+V, respectively. Moreover, we classified all patients into four different histological grades, 14 (7.1%) in G1, 20 (10.2%) in G2, 114 (58.2%) in G3+G4 and 17 (8.7%) in borderline tumor, but an exception of 31 cases (15.8%) were into the groups of “undetermined” due to the lack of information.

### Association between the SNPs of BER-related genes and risk of ovarian cancer

Genotype distributions of these 19 SNPs are shown in** Table 1**. All these genotype distributions among the controls conformed to the HWE except *FEN1* rs174538. Nevertheless, our results verified *PARP* rs8679 and *hOGG1* rs293795 GG/AG were associated with the decreased risk of ovarian cancer under dominant model (adjusted OR=0.39, 95% CI=0.17-0.90, *P*=0.026; adjusted OR=0.36, 95% CI=0.13-0.99, *P*=0.049, respectively). And, the increased risk of ovarian cancer was observed in the participants who carried rs4796030 AA genotype compared with the genotype AC/CC (adjusted OR=1.54, 95% CI=1.01-2.35, *P*=0.046) under recessive model. However, other genetic associations with ovarian cancer risk were not discovered in the present study.

### Stratification analysis

In order to further explore these significant genetic associations existing in which kinds of ovarian cancer patients, we performed a stratification analysis by age, BMI, menarche, menopause and number of pregnancies status, as well as some clinic-related factors like pathologic classification, histology grade, FIGO stage, tumor size and anatomic neoplasm subdivision (**Table 2**). Compared to the *PARP1* rs8679 AA genotype, the protective effect of AG/GG genotypes were more predominant in the patients with BMI < 24 (adjusted OR=0.27, 95% CI=0.08-0.96, *P*=0.043), menarche age ≤ 14 (adjusted OR=0.09, 95% CI=0.01-0.65, *P*=0.017) and serous carcinoma (adjusted OR=0.36, 95% CI=0.14-0.98, *P*=0.045). Moreover, we found the *LIG3* rs4796030 CC genotype carriers further increased the risk of ovarian cancer in the groups of BMI ≥ 24 (adjusted OR=2.28, 95% CI=1.14-4.58, *P*=0.021), clinical stages III/IV (adjusted OR=1.93, 95% CI=1.14-3.26, *P*=0.015) and tumor at the left (adjusted OR=2.33, 95% CI=1.34-4.06, *P*=0.003) when compared with the AA/AC genotype carriers. Unfortunately, stratification analysis could not explain the protective effect of *hOGG1* rs293795 polymorphism on which subgroup of ovarian cancer patients.

## Discussion

The evidence derived from numerous studies had testified the important role of BER in the prevention of mutation accumulation 23, 24. According to different base lesions, BER can trigger corresponding subways and assemble a series of repair complex at the site of DNA lesions 25. Compared with other DNA repair system, the core components of BER are quite conserved across most species. As is mentioned above, the whole repair processes are simplified as three distinct phases, and the completion of each step is based on the scaffold protein of XRCC1/LIG3 and PARP1 26. hOGG1, one of the most important bi-functional glycosylases, is initiated by some oxidative base lesions 13. The study reported by Xie *et al.* found that the *hOGG1* deficiency animals would increase tumor predisposition, especially for the lung and ovarian tumors and lymphomas 27. Similarly, with the increasing of spontaneous mutant frequencies in the *APE1* heterozygous mice, the organism also suffered many deleterious effects brought by oxidative stress, including cancer 28, 29. Although FEN1 was the only one endonuclease included in the present study, its potential role in cancer development was fully demonstrated by a heterozygous animal model 30.

Numerous investigations have tried to uncover the genetic association of ovarian cancer in these six genes; however, no firm conclusions could be drawn from them. For example, *hOGG1* rs1052133 as one of the hottest spots in the recent years has been researched extensively in ovarian cancer. According to the studies of Michalska and Chen, *hOGG1* rs1052133 C>G polymorphism was considered as an unfavorable factor for the susceptibility of ovarian cancer 31, 32, but a totally different result was discovered in our research. Arc and reached the same conclusion with us and suggested that there was no association between both of them 33. Likewise, a negative association was observed between *XRCC1* rs25487 G>A and ovarian cancer risk in Serbian women 34, while this finding was unable to repeat in another study by Khokhrin and ours 35. In fact, these contradictory conclusions were caused by several factors, of which the relatively limited sample size and different genetic backgrounds a probably the most critical reasons.

In this current hospital-based case-control study, we not only explored the impact of 19 potentially functional SNPs in six BER-related genes on ovarian cancer risk, but also conducted an analysis of gene-environmental interactions in a certain number of cases and controls from two different institutions.

The genotyping results indicated that the *PARP1* rs8679 and *hOGG1* rs293795 A>G polymorphisms were associated with the decreased ovarian cancer risk under a dominant model, while *LIG3* rs4796030 A>C polymorphism with an increased ovarian cancer risk under a recessive model. Additionally, we noticed that the protective effect of *PARP1* rs8679 A>G polymorphism was more pronounced among the groups of BMI < 24, menarche age ≤ 14 and serous carcinoma. Moreover, the negative effect of *LIG3* rs4796030 A>C polymorphism significantly associated with the patients in the groups of BMI ≥ 24, clinical stages III/IV and tumor at the left. The preliminary results obtained here indicated that compared with patients of BMI ≥ 24, patients of BMI < 24 had a more prominent protective effect of genetic variation, and their negative effects were relatively small. These results showed that healthy lifestyle also played an important role in the development of cancer. The role of genetic variation was also related to the pathological type and clinical stage of cancer.

Although we found two novel related SNPs associating with the risk of ovarian cancer, some inherent limitations still should be listed to discussion. Firstly, the limited number of cases and controls would cause a deficiency of statistical power to some extent. Secondly, many relative risk factors of ovarian cancer, such as oral contraceptive, CA125 and breast feeding, are not fully considered due to lack of individual information. Thirdly, only 19 SNPs of six BER-related genes were investigated in the present study, which was insufficiently to explain the biological relationship of BER with the risk of ovarian cancer. Fourthly, we noticed that the genotype frequencies of *FEN1* rs174538 G>A deviate from HWE, which indicates that our research may exist the problem sampling bias to some extent. Finally, these new findings in our research were not confirmed *in vitro* and *in vivo* experiences, and the survival analysis for SNPs was not explored further.

In conclusion, our study suggested that the *PARP1* and *hOGG1* polymorphisms might correlate to ovarian cancer susceptibility. However, more comprehensive studies with larger independent cohorts should be provided to verify the relationship between these significant genetic variations in *PARP1* and *hOGG1* gens and ovarian cancer risk.

## Supplementary Material

Supplementary tables.

## Figures and Tables

**Table 1:** Association between polymorphisms in base excision repair pathway gene and ovarian cancer risk

Gene	SNP	Allele		Case (N=196)			Control (N=272)			Adjusted OR^a^	*P^a^*	Adjusted OR^b^	*P*^b^	HWE
		A	B	AA	AB	BB	AA	AB	BB	(95% CI)		(95% CI)		
*PARP1*	rs2666428	T	C	129	49	7	171	87	13	0.73 (0.49-1.09)	0.126	0.80 (0.31-2.05)	0.637	0.653
*PARP1*	rs8679	A	G	177	8	0	241	26	1	**0.39 (0.17-0.90)**	**0.026**	-	-	0.740
*hOGG1*	rs1052133	G	C	78	87	29	110	115	47	1.03 (0.71-1.51)	0.870	0.84 (0.50-1.39)	0.486	0.079
*hOGG1*	rs159153	T	C	160	32	1	221	46	5	0.95 (0.58-1.54)	0.823	0.28 (0.03-2.46)	0.252	0.164
*hOGG1*	rs293795	A	G	183	5	0	253	18	1	**0.36 (0.13-0.99)**	**0.049**	-	-	0.279
*FEN1*	rs174538	G	A	45	143	0	73	199	0	1.17 (0.76-1.79)	0.484	-	-	**<0.001**
*FEN1*	rs4246215	G	T	46	96	52	74	135	63	1.22 (0.80-1.88)	0.361	1.26 (0.82-1.93)	0.296	0.925
*APEX1*	rs1130409	T	G	64	100	29	91	123	54	1.05 (0.71-1.57)	0.801	0.72 (0.43-1.18)	0.190	0.293
*APEX1*	rs1760944	T	G	65	89	38	104	122	46	1.18 (0.80-1.74)	0.412	1.18 (0.73-1.91)	0.501	0.321
*APEX1*	rs3136817	T	C	158	33	3	224	43	5	1.08 (0.67-1.75)	0.754	0.85 (0.20-3.64)	0.822	0.095
*LIG3*	rs1052536	C	T	92	85	15	131	116	21	1.04 (0.71-1.51)	0.847	1.02 (0.51-2.05)	0.958	0.502
*LIG3*	rs3744356	C	T	188	3	1	261	10	0	0.56 (0.17-1.82)	0.331	-	-	0.757
*LIG3*	rs4796030	A	C	77	55	61	88	117	62	0.73 (0.49-1.08)	0.112	**1.54 (1.01-2.35)**	**0.046**	0.060
*XRCC1*	rs1799782	G	A	99	75	19	146	105	18	1.11 (0.77-1.62)	0.570	1.53 (0.78-3.01)	0.218	0.881
*XRCC1*	rs25487	C	T	89	83	21	146	97	24	1.43 (0.98-2.08)	0.063	1.31 (0.70-2.45)	0.395	0.182
*XRCC1*	rs25489	G	A	139	50	5	215	50	7	1.48 (0.96-2.28)	0.074	0.99 (0.31-3.21)	0.985	0.059
*XRCC1*	rs2682585	G	A	146	44	3	205	60	3	1.06 (0.68-1.64)	0.803	1.49 (0.29-7.62)	0.634	0.547
*XRCC1*	rs3810378	G	C	102	70	23	147	101	24	1.10 (0.76-1.60)	0.624	1.45(0.79-2.66)	0.236	0.273
*XRCC1*	rs915927	T	C	159	33	1	213	56	3	0.76 (0.47-1.22)	0.261	0.49 (0.05-4.87)	0.543	0.749

OR, odds ratio; CI, confidence interval. HWE, Hardy-Weinberg equilibrium. The results were in bold, if the 95% CI excluded 1 or *P*-values less than 0.05. a: Adjusted for age, BMI, menarche, menopause, number of pregnancies for dominant model. b: Adjusted for age, BMI, menarche, menopause, number of pregnancies for recessive model.

**Table 2:** Stratification analysis of base excision repair pathway gene variant genotypes with ovarian cancer risk

Variables	*PARP1* rs8679 (cases/controls)		AOR (95% CI)^a^	*P*^a^	*hOGG1* rs293795 (cases/controls)		AOR (95% CI)^a^	*P*^a^	*LIG3* rs4796030 (cases/controls)		AOR (95% CI)^a^	*P*^a^
	AA	AG/GG			AA	AG/GG			AA/AC	CC		
**Age**												
< 51	86/112	5/13	0.50 (0.17-1.46)	0.205	88/116	3/10	0.40 (0.11-1.48)	0.168	70/100	25/24	1.49 (0.79-2.82)	0.222
≥ 51	91/129	3/14	0.30 (0.09-1.09)	0.067	95/137	2/9	0.32 (0.07-1.52)	0.151	62/105	36/38	1.60 (0.92-2.79)	0.094
**BMI**												
**< 24**	119/162	3/15	**0.27 (0.08-0.96)**	**0.043**	122/167	3/14	0.29 (0.08-1.04)	0.058	94/135	35/41	1.23 (0.73-2.07)	0.445
**≥ 24**	58/79	5/12	0.57 (0.19-1.70)	0.312	61/86	2/5	0.56 (0.11-3.00)	0.502	38/70	26/21	**2.28 (1.14-4.58)**	**0.021**
**Menarche**												
≤ 14	95/145	1/18	**0.09 (0.01-0.65)**	**0.017**	97/156	1/11	0.15 (0.02-1.15)	0.068	73/129	28/34	1.46 (0.82-2.59)	0.202
> 14	82/96	7/9	0.91 (0.33-2.55)	0.859	86/97	4/8	0.56 (0.16-1.94)	0.363	59/76	33/28	1.52 (0.83-2.79)	0.178
**Menopause**												
no	87/110	4/12	0.42 (0.13-1.35)	0.146	90/115	2/8	0.32 (0.07-1.54)	0.155	70/100	25/21	1.70 (0.88-3.28)	0.112
yes	90/131	4/15	0.39 (0.13-1.21)	0.102	93/138	3/11	0.41 (0.11-1.49)	0.174	62/105	36/41	1.49 (0.86-2.57)	0.155
**Number of pregnancies**												
≤ 1	63/78	2/9	0.28 (0.06-1.32)	0.107	66/84	0/4	-	-	50/69	19/17	1.54 (0.73-3.26)	0.257
> 1	114/163	6/18	0.48 (0.18-1.24)	0.128	117/169	5/15	0.48 (0.17-1.36)	0.168	82/136	42/45	1.55 (0.94-2.56)	0.088
**Clinical stages**												
I/II	87/241	3/27	0.30 (0.09-1.03)	0.056	90/253	2/19	0.33(0.07-1.44)	0.140	63/205	33/62	**1.93 (1.14-3.26)**	**0.015**
III/IV	90/241	5/27	0.52 (0.19-1.41)	0.197	93/253	3/19	0.40 (0.11-1.39)	0.147	69/205	28/62	1.27(0.74-2.17)	0.387
**Pathology**												
Serous carcinoma	119/241	5/27	**0.36 (0.14-0.98)**	**0.045**	121/253	4/19	0.41 (0.13-1.25)	0.118	87/205	43/62	1.58 (0.98-2.53)	0.058
Mucinous carcinoma	25/241	0/27	-	-	26/253	0/19	-	-	16/205	10/62	2.34 (0.98-5.57)	0.055
Clear cell carcinoma	15/241	0/27	-	-	15/253	0/19	-	-	12/205	3/62	0.94 (0.25-3.52)	0.922
Others	18/241	3/27	1.52 (0.41-5.67)	0.533	21/253	1/19	0.65 (0.08-5.18)	0.680	17/205	5/62	0.95 (0.33-2.74)	0.922
**Anatomic neoplasm subdivision**												
Left	67/241	5/27	0.67 (0.25-1.81)	0.425	68/253	4/19	0.81 (0.26-2.50)	0.718	43/205	30/62	**2.33 (1.34-4.06)**	**0.003**
Right	49/241	0/27	-	-	51/253	0/19	-	-	43/205	9/62	0.75(0.34-1.67)	0.488
Bilateral	61/241	3/27	0.45 (0.13-1.54)	0.201	64/253	1/19	0.17 (0.02-1.37)	0.097	46/205	22/62	1.49 (0.82-2.72)	0.189
**Tumor size**												
< 5 cm	23/241	1/27	0.41 (0.05-3.17)	0.392	25/253	0/19	-	-	20/205	7/62	1.15 (0.45-2.91)	0.770
5-10 cm	69/241	3/27	0.39 (0.11-1.33)	0.132	68/253	4/19	0.78 (0.25-2.43)	0.672	49/205	24/62	1.58 (0.89-2.83)	0.119
> 10 cm	85/241	4/27	0.42 (0.14-1.25)	0.121	90/253	1/19	0.15 (0.02-1.16)	0.069	63/205	30/62	1.62 (0.96-2.74)	0.074
**Grade**												
G1	13/241	0/27	-	-	14/253	0/19	-	-	9/205	5/62	1.20 (0.68-7.10)	0.189
G2	18/241	1/27	0.54 (0.07-4.29)	0.558	20/253	0/19	-	-	11/205	9/62	2.61 (1.00-6.84)	0.051
G3&G4	100/241	6/27	0.55 (0.22-1.39)	0.206	104/253	4/19	0.47 (0.15-1.44)	0.187	81/205	30/62	1.14(0.68-1.91)	0.618
Borderline tumor	17/241	0/27	-	-	16/253	0/19	-	-	8/205	9/62	5.30 (1.82-15.43)	0.002
Undetermined	29/241	1/27	0.30 (0.04-2.33)	0.250	29/253	1/19	0.61 (0.08-4.84)	0.639	23/205	8/62	0.77 (0.35-1.69)	0.509

CI, confidence interval; AOR, adjusted odds ratio. The results were in bold, if the 95% CI excluded 1 or *P* values less than 0.05. a: Obtained in logistic regression models with adjustment for age, BMI, menarche, menopause, number of pregnancies.
