# Supplementary material for: The Association of Polymorphisms in Base Excision Repair Genes with Ovarian Cancer Susceptibility in Chinese Women: A Two-Center Case-Control Study
Source: J Cancer. 2021 Jan 1;12(1):264–9. doi: 10.7150/jca.49925 (PMC7738827; doi:10.7150/jca.49925)
Supplement: Supplementary file 1 — Supplementary tables. [file jcav12p0264s1.pdf]

Supplemental Table 1. Potential functional polymorphisms in base excision repair pathway genes as predicted by SNPinfo online software

rs	Chr	Allele	Position	TFBS	Splicing (ESE or ESS)	Splicing (abolish domain)	miRNA (miRanda)	miRNA (Sanger)	nsSNP	Nearby Gene	Distance (bp)	Allele	Asian	CHB
rs2666428	1	A/C	224656332	--	Y	--	--	Y	--	*PARP1*	41317‖6092	T	0.833	0.804
rs8679	1	C/A	224615177	--	--	--	Y	Y	--	*PARP1*	162‖47247	A	0.934	0.958
rs1052133	3	G/A	9773773	--	Y	--	Y	--	Y	*OGG1*	8068‖9569	C	0.489	0.5
rs159153	3	T/C	9764875	Y	--	--	--	--	--	*BRPF1‖OGG1*	-176‖-830	T	0.91	0.893
rs293795	3	T/G	9774113	--	--	--	Y	Y	--	*OGG1/CAMK1*	8408/82‖9229/12548	A	0.939	0.952
rs174538	11	T/G	61316657	Y	Y	--	--	--	--	*C11orf10*	3476‖4	G	0.644	0.679
rs4246215	11	G/A	61320875	Y	--	--	Y	--	--	*FEN1*	4149‖411	G	0.672	0.679
rs1130409	14	C/G	19994994	--	Y	--	--	--	Y	*APEX1*	1864‖772	T	0.611	0.524
rs1760944	14	C/G	19992989	Y	Y	--	--	--	--	*OSGEP*	7933‖49	G	0.561	0.429
rs3136817	14	G/T	19994274	Y	--	--	--	--	--	*APEX1*	1144‖1492	T	--	0.866
rs1052536	17	G/T	30355688	--	--	--	Y	Y	--	*LIG3*	24037‖513	C	0.741	0.711
rs3744356	17	C/G	30337242	--	Y	--	--	--	Y	*LIG3*	5591‖18959	C	0.942	0.976
rs4796030	17	T/C	30354263	--	Y	Y	Y	Y	--	*LIG3*	22612‖1938	C	0.429	0.423
rs1799782	19	G/A	48749414	--	Y	--	--	--	Y	*XRCC1*	10110‖22141	G	0.739	--
rs25487	19	C/T	48747566	--	Y	--	--	--	Y	*XRCC1*	8262‖23989	C	0.724	0.747
rs25489	19	C/T	48748252	--	Y	--	--	--	Y	*XRCC1*	8948‖23303	C	1	0.904
rs2682585	19	A/G	48773128	Y	--	--	--	--	--	*XRCC1‖LOC390940*	-1573‖-105	G	0.878	0.877
rs3810378	19	C/G	48773441	Y	--	--	--	--	--	*LOC390940*	208‖4670	G	0.748	0.75
rs915927	19	T/C	48749067	--	Y	--	--	--	--	*XRCC1*	9763‖22488	T	0.891	--

TFBS, transcription factor binding sites; ESE, exon splicing enhancer; ESS, exon splicing silencer; CHB, Han Chinese in Beijing, China.

Supplemental Table 2. Frequency distribution of selected variables for ovarian cancer cases and cancer-free controls

Characteristic	Case (n=196)	Control (n=272)	*P* [a]
Age	No. (%)	No. (%)	0.570
<51	96 (49.0%)	126 (46.3%)	
≥51	100 (51.0%)	146 (53.7%)	
BMI			0.947
<24	131 (66.8%)	181 (66.5%)	
≥24	65 (33.2%)	91 (33.5%)	
Menarche			0.072
≤14	104 (53.1%)	167 (61.4%)	
>14	92 (46.9%)	105 (38.6%)	
Menopause			0.421
no	96 (49.0%)	123 (45.2%)	
yes	100 (51.0%)	149 (54.8%)	
Number of pregnancies			0.448
≤1	70 (35.7%)	88 (32.4%)	
>1	126 (64.3%)	184 (67.7%)	
Pathology			
Serous carcinoma	132 (67.4%)		
Mucinous carcinoma	27 (13.8%)		
Clear cell carcinoma	15 (7.7%)		
Others	22 (11.2%)		
FIGO stage			
I+II	96 (49.0%)		
III+IV	100 (51.0%)		
Anatomic neoplasm subdivision			
Left	73 (37.2%)		
Right	53 (27.0%)		
Bilateral	70 (35.7%)		
Tumor size			
<5cm	27 (13.8%)		
5-10cm	74 (37.8%)		
>10cm	95 (48.5%)		
Grade			
G1	14 (7.1%)		
G2	20 (10.2%)		
G3&G4	114 (58.2%)		
Borderline tumor	17 (8.7%)		
undetermined	31 (15.8%)		

[a] Two-sided χ^2 test for distributions between ovarian cancer cases and cancer-free controls.
